# Predictive Modelling of Alkali-Slag Cemented Tailings Backfill Using a Novel Machine Learning Approach

**DOI:** 10.3390/ma18061236

**Published:** 2025-03-11

**Authors:** Haotian Pang, Wenyue Qi, Hongqi Song, Haowei Pang, Xiaotian Liu, Junzhi Chen, Zhiwei Chen

**Affiliations:** 1 Key Laboratory of Xinjiang Coal Resources Green Mining, Ministry of Education, Xinjiang Institute of Engineering, Urumqi 830023, China; 18852142412@163.com; 2 Hebei Province Engineering Research Center for Harmless Synergistic Treatment and Recycling of Municipal Solid Waste, Yanshan University, Qinhuangdao 066004, China; p18682824506@163.com (H.P.); 15589753101@163.com (H.S.); pppppphw@163.com (H.P.); liuxiaotian200010@163.com (X.L.); 3 Shicao Village Coal Mine, State Energy Group Ningxia Coal Industry Co., Ltd., Yinchuan 750400, China; czw_lucky@163.com

**Keywords:** cemented tailings backfill, machine learning, solid waste treatment, mechanical performance prediction, dynamic prediction

## Abstract

This study utilizes machine learning (ML) techniques to predict the performance of slag-based cemented tailings backfill (CTB) activated by soda residue (SR) and calcium carbide slag (CS). An experimental database consisting of 240 test results is utilized to thoroughly evaluate the accuracy of seven ML techniques in predicting the properties of filling materials. These techniques include support vector machine (SVM), random forest (RF), backpropagation (BP), genetic algorithm optimization of BP (GABP), radial basis function (RBF) neural network, convolutional neural network (CNN), and long short-term memory (LSTM) network. The findings reveal that the RBF and SVM models demonstrate significant advantages, achieving a coefficient of determination (*R*^2^) of approximately 0.99, while the *R*^2^ for other models ranges from 0.86 to 0.98. Additionally, a dynamic growth model to predict strength is developed using ML techniques. The RBF model accurately predicts the time required for filling materials to reach a specified strength. In contrast, the BP, SVM, and CNN models show delays in predicting this curing age, and the RF, GABP, and LSTM models tend to overestimate the strength of the filling material when it approaches or fails to reach 2 MPa. Finally, the RBF model is employed to perform coupling analysis on filling materials with various mix ratios and curing ages. This analysis effectively predicts the changes in filling strength over different curing ages and raw material contents, offering valuable scientific support for the design of filling materials.

## 1. Introduction

With the ceaseless development and utilization of mineral resources, the generation of solid waste, such as industrial waste residue and tailings, has increased sharply. Effectively treating bulk industrial solid waste has become one of the critical issues worldwide [1,2]. Cementing–filling technology mixes tailings with other components like hydraulic binders and water to form a filling body with certain fluidity and solidification characteristics. When utilized to fill underground goaf, this technology not only ensures the stability of underground mining areas [3,4,5,6] but also eliminates the discharge of surface tailings waste, providing a safe, reliable, environmentally friendly, and sustainable approach to managing solid waste generated in mines.

However, in the previous design process of cementing–filling materials, researchers mainly adopted empirical intervention and basic experiments. They used key filling parameters of similar mines for reference and performed optimization by combining indoor measurement results such as fluidity by slump meter and unconfined compressive strength by mechanical testing instruments. This method has disadvantages such as a high testing cost, long time consumption, and lengthy sample preparation cycles [7,8,9]. Current research mainly focuses on the impact of single design parameters on the performance of cemented paste backfill (CPB), such as the proportions of binder and activator, liquid–solid ratio, particle size distribution of tailings, cementitious material/tailings ratio, and curing age [10,11,12,13,14]. Due to the complex composition and numerous influencing factors of filling slurry, conflicts between these key parameters and indicators may arise, making it more difficult to obtain a globally optimal solution. For instance, enhancing the fluidity of the slurry to prevent pipeline blockage may reduce the yield stress of CPB and weaken its long-term stability. Therefore, optimizing CPB performance parameters is essentially a multi-objective optimization problem. The lack of an integrated design framework exacerbates this challenge [15,16], highlighting the urgency of establishing an economical and rapid method for predicting CPB’s mechanical performance.

Accurately predicting the performance of CPB is a challenging task. The development of different engineering models using machine learning (ML) has become increasingly significant [17,18,19]. Chendi et al. [20] used six integrated learning models to predict the early-stage compressive strength of cemented phosphogypsum (PG) backfill soil. Among the six models, the extreme gradient boosting (XGBoost) model performed the best, with an *R*^2^ of 0.990, a root mean squared error (RMSE) of 0.050, and a mean absolute error (MAE) of 0.034. Chathuranga et al. [21] used two integrated ML and a single ML method to predict the unconfined compressive strength (UCS) of alkali-activated slag-based CPB. The results indicated that the integrated learning method was superior to the single learning method in predicting UCS and found that the water-to-cement ratio and curing time were the most critical input parameters in the model. Yafei et al. [22] used the sparrow search algorithm to construct a model for predicting CPB strength with curing time, slurry concentration, sand-to-cement ratio, and curing temperature as input variables and UCS as the output variable. Compared with algorithms such as BP, its *R*^2^ value increased to 0.9979. Weijun et al. [23] used four ML techniques to predict the compressive strength of a titanium tailings-based backfilling body. The study indicated that the whale optimization–random forest integrated algorithm achieved good prediction results (*R*^2^ = 0.975). In summary, the use of computer modeling technology and ML algorithms can more accurately predict the performance of backfilling materials. However, in the prediction of the mechanical properties of multi-component solid waste-based filling materials, the depth of research using the ML technique is still insufficient. It is urgent to develop new ML models to guide the design of SR-activated CPB.

Accordingly, this study aims to comprehensively evaluate the predictive effects of different ML techniques on the performance of filling materials, determine the strength threshold (UCS > 2 MPa) according to actual engineering needs, and output the corresponding curing age. Section 2 explains the key points and parameter optimization for each ML technique. In Section 3, the performances of different ML techniques in predicting fluidity and strength are introduced and compared. In Section 4, different ML techniques are utilized to predict the growth in the strength of filling materials, and RBF-based models with different proportions and curing ages are established, which have deeper guiding significance for practical engineering projects.

## 2. Methods

### 2.1. Data Collection

According to the “Test Method for Fluidity of Cement Mortar” (GB/T 2419-2005 [24]), the slump of the newly mixed cementitious filling slurry was tested using a conical mold. The diameters of the newly mixed filling slurry in two vertical directions were measured three times using a caliper, and the average value was taken as the test result. Referring to the “Standard for Test Method of Mechanical Properties on Ordinary Concrete” (GB/T 50081-2002 [25]), the cementitious filling specimens were cured for 3, 7, 14, and 28 days, respectively. The compressive strengths of the specimens were tested using an MTS electro-hydraulic servo instrument, and the average values were taken as the test results. The input parameters were mass concentration, slag content, and proportions of activator/binder and SR, while the output parameters were the slump of freshly mixed slurry and the compressive strength of CPB. The experiment tested the average values and variances of fluidity and strengths of 240 test specimens from 20 small groups consisting of 4 large groups. The test database is shown in Table 1. The obtained data were modeled using the ML method to evaluate liquidity and compressive strength. It is well known that the dispersion and distribution of available data can affect the performance of artificial intelligence models. Finally, the data provided by all models were screened according to the intervals of variables, ensuring that the CPB specimen met actual engineering requirements (F > 180 mm, UCS > 2 MPa).

### 2.2. Overview of Learning Algorithms

This study utilized seven representative ML algorithms, namely SVM, RF, ANN (BP, GABP, RBF), CNN, and RNN (LSTM), to predict the performance of SR-activated CPB specimens. These algorithms have been elaborated in detail in the references [26,27,28,29], so this article does not repeat the description of these algorithms.

#### 2.2.1. Support Vector Machine

SVM is an ML algorithm utilized to solve binary classification problems [30]. The algorithm adopted the optimal boundary to segment the characteristic space input. The boundary was defined as a hyperplane when the characteristic space was limited to 2-D space. Assuming that $D_{0}$ and $D_{1}$ are two sets of points in a *n*-dimensional Euclidean space, if there exists an *n*-dimensional vector w and a real number *b* such that all points belonging to *D*_0_ have $wx_{i}+b>0$, while all points belonging to *D*_1_ have $wx_{j}+b<0$, then $D_{0}$ and $D_{1}$ are linearly separable [31]. When expanding from two-dimensional to multi-dimensional space, the wx+b=0 that completely separates *D*_0_ and *D*_1_ is a hyperplane. The linear equation of any hyperplane is as follows:

(1) $$w^{T}x+b=0$$

Given the parameters of the kernel function, support vector regression (SVR) must find a hyperplane that minimizes the sum of distances between all data points and the hyperplane. Its standard form is as follows:

(2) $$\underset{w,b,\xi ,\xi^{*}}{\min}\frac{1}{2}w^{T}w+C\sum_{i=1}^{N}\left(\xi_{i}+\xi_{i}^{*}\right)$$

Constraints:

(3) $$f(x_{i})-y_{i}\leq \varepsilon +\xi_{i}\;y_{i}-f(x_{i})\leq \varepsilon +\xi_{i}^{*}\;\xi_{i},\xi_{i}^{*}\geq 0,i=1,2,\ldots ,N$$

where C>0 and ε>0; f(x)=wx+b is the final model function; $\xi^{*}$ is the difference between the sample point *y* and the projection of the sample point below the lower edge of the isolation band to the lower edge of the isolation band.

#### 2.2.2. Random Forest

The RF algorithm is a method that combines multiple decision trees [20], which mitigates the over-fitting problem encountered when using a single decision tree, thereby reducing the noise sensitivity of the training set. The algorithm initially employs Bootstrap sampling to determine the sample points for subsequent prediction from the original training set, ensuring that each sample point has the same size as the original training set. RF then counts all decision trees in the forest and votes on the predicted values to produce the final result [32,33].

#### 2.2.3. Artificial Neural Network

(a) Backpropagation

BP is a multi-layer feed-forward neural network characterized by forward transmission of signals and BP of error. If the expected output is not obtained, it will switch to BP mode and adjust the network weights and thresholds based on the prediction error. The BP neural network’s predicted output can continuously approach the expected output [34]. Let $p_{i}$ and $p_{j}$ be neuron in the input layer; $Net_{i}$ be a neuron in the hidden layer; $w_{i}$ and $w_{j}$ be the initial weight from the input layer to the hidden layer; $w_{k}$ and $w_{l}$ be the initial weight from the hidden layer to the output layer; and $b_{i}$ and $b_{j}$ be the bias from the input layer to the hidden layer and the bias from the hidden layer to the output layer, respectively [35]. Therefore, the formula that governs the relation from the input layer to the hidden layer is as follows:

(4) $$Net_{i}=w_{i}\times p_{i}+w_{j}\times p_{j}+b_{i}$$

Upon obtaining output values $Out_{Ni}$ and $Out_{Nj}$, the relationship from the hidden layer to the input layer is as follows:

(5) $$Net_{i}=w_{k}\times Out_{Ni}+w_{l}\times Out_{Nj}+b_{j}$$

**Figure 1:**
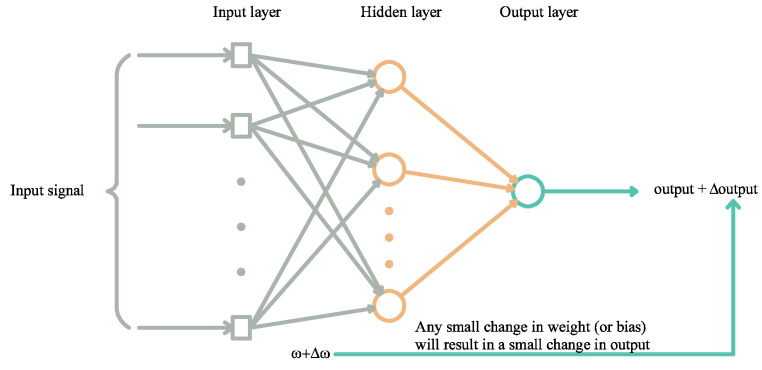
ANN model process diagram.

Once the final output values $Out_{i}$ and $Out_{j}$ are obtained, the error propagates back if the error compared to the actual value is greater than the specified threshold. Subsequently, the weights are updated and recalculated, and the iterations are conducted continuously until the error value falls below the threshold (see Figure 1) [22].

(b) Genetic algorithm optimization of BP

The reliability and stability of the model are affected by the initial random parameters during the training process. However, in most cases, the random initial parameter values of the BP model are not the optimal starting point. Under these conditions, the genetic algorithm (GA) demonstrates its advantages. As a heuristic algorithm, GA possesses strong global search capability and can optimize the initial weights and thresholds [36]. The optimization parameters are the initial weights and thresholds of the BP neural network [37,38].

(c) Radial basis function neural network

As a three-layer feed-forward neural network structure with only one hidden layer, the most distinct difference between RBF and feed-forward networks is that the transformation function of the hidden layer is a Gaussian function with local response. Using RBF as the “base” of the hidden unit to form the hidden layer space, the input vector can be directly mapped to the hidden space without the need for weight connections [39]. The mapping from the hidden layer space to the output space is linear, meaning the network’s output is a linear weighted sum of the outputs of hidden units. In this structure, the function of the hidden layer is to map the vector from the low dimensional *p* to the high dimensional *h* [40,41]. The Gaussian kernel function of the RBF neural network is as follows:

(6) $$G_{i}(X)=\exp(-\frac{||X-C_{i}|{|}^{2}}{2\sigma_{i}^{2}})$$

where *X* denotes an n-dimensional input vector; *C* denotes the center value of the basis function; σ is a standardized constant for the width of the center point of the basis function.

Select h centers for k-means clustering, and the network output of RBF is:

(7) $$f(x)=\frac{\sum_{i=1}^{n_{h}}w_{i}G_{i}(X)}{\sum_{i=1}^{n_{h}}G_{i}(X)}$$

where $w_{i}$ denotes the weight from the hidden layer to the output layer; $G_{i}(X)$ is an RBF from the input layer to the hidden layer.

#### 2.2.4. Convolutional Neural Network

**Figure 2:**
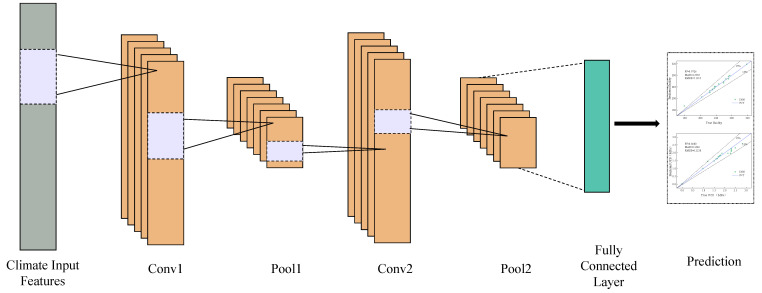
CNN model process diagram.

As a feed-forward neural network with a convolutional structure, CNN mainly consists of convolution layers, pooling layers, and fully connected layers and can effectively reduce the memory occupied by deep networks [42,43]. Figure 2 shows the overall architecture of CNN, consisting of two convolution layers (Conv1 and Conv2), two pooling layers (Pool1 and Pool2), and a fully connected layer [44]. The parameters of the convolution layer and fully connected layer will be trained as the gradient decreases, effectively reducing the number of network parameters and alleviating the model’s over-fitting problem. The fully connected layer of the CNN model is modified to output only continuous values, and the regression loss function is utilized to train the network to accomplish regression prediction [45,46]. At this point, CNN is utilized to map a characteristic space.

(8) $$\left\{\begin{array}{cccc} \delta_{1}^{1} & \delta_{1}^{2} & \cdots & \delta_{1}^{n}\\ \delta_{2}^{1} & \delta_{2}^{2} & \cdots & \delta_{2}^{n}\\ \vdots & \vdots & & \vdots \\ \delta_{m}^{1} & \delta_{m}^{2} & \cdots & \delta_{m}^{n} \end{array}\right\}\to \left\{\begin{array}{c} r_{1}\\ r_{2}\\ \vdots \\ r_{m} \end{array}\right\}$$

where $\delta_{i}^{j}$ is the characteristics input; $r_{i}$ is the predicted values, 1≤i≤m, 1≤j≤n; m is the length of the dataset; and n is the number of characteristics input.

#### 2.2.5. Recurrent Neural Network

**Figure 3:**
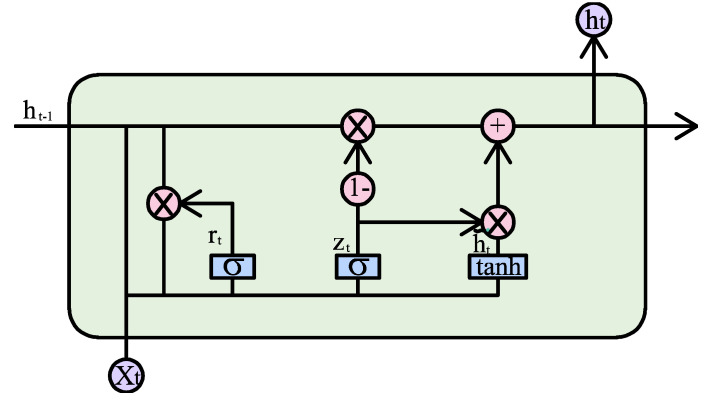
RNN model process diagram.

RNN is a type of time-recursive neural network suitable for processing and predicting important events with relatively long intervals and delays in time series. LSTM networks were proposed to solve the “gradient vanishing” problem that exists in RNNs [47]. Based on the new input and the output of the previous moment, it is decided to forget some specific memories, integrate them into a separate vector, and multiply them in the unit state through the *sigmoid* neural layer [48,49]. This operation is undertaken by the “forget gate”, and the formula is as follows:

(9) $$f_{t}=\sigma (W_{f}\cdot [h_{t-1},x_{t}]+b_{f})$$

where *f_t_* is the output vector of the *sigmoid* neural layer. Firstly, the effective information from the vector is extracted using the *tanh* function layer, then it is controlled using the *sigmoid* function, and finally, it is output as follows:

(10) $$i_{t}=\sigma (W_{i}\cdot [h_{t-1},x_{t}]+b_{i})\;o_{t}=\sigma (W_{o}\cdot [h_{t-1},x_{t}]+b_{o})\;{\tilde{C}}_{t}=\tanh(W_{c}\cdot [h_{t-1},x_{t}]+b_{c})$$

where *f_t_* denotes the forget gate; *i_t_* is the input gate; *o_t_* is the output gate; ${\tilde{C}}_{t}$ is the generated input value vector; *W* is the parameter matrix; *x* is the input; *h* is the state vector; and *b* is the bias term (see Figure 3).

### 2.3. Dataset Preprocessing and Analysis

#### 2.3.1. Data Preprocessing

The range of initial data will result in the gradient directions at most positions not being the optimal search direction. Therefore, using gradient descent to seek the optimal solution requires multiple iterations to converge. Additionally, to avoid the decrease in evaluation accuracy of the model caused by significant differences in data magnitude, the data preprocessing process adopts 0–1 normalization, as shown in Equation (11).

(11) $$x^{*}=\frac{x-x_{\min}}{x_{\max}-x_{\min}}$$

#### 2.3.2. Data Analysis

The model is trained using 80% of the dataset and adjusted through the validation set to ensure its generalization performance on unseen data. Subsequently, 20% of an independent testing database is utilized to evaluate the reliability and predictive performance of the model [50].

### 2.4. Model Construction

**Figure 4:**
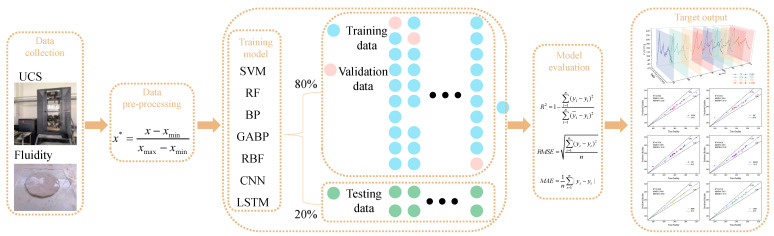
Research method flow chart.

MATLAB 2022B is used to develop ML models such as SVM, RF, BP, GABP, RBF, CNN, LSTM, etc. The development approach is divided into five stages: data collection, data preprocessing, model training, model evaluation, and target output, as shown in Figure 4.

#### 2.4.1. Tuning of Hyperparameters

The hyperparameters of a model play an important role in its predictive accuracy since they determine the learning speed and recognition method of the model [51,52]. This study uses a grid search CV function for automatically tuning and optimizing hyperparameters [53,54,55].

#### 2.4.2. K-Fold Cross-Validation

K-fold cross-validation has often been used previously to enhance the reproducibility of results predicted by the model [56,57]. This study used 10-fold cross-validation. Concretely, the validation phase divided 80% of training sets into ten groups, with one group being retained to validate and returned 10 target variables. The model performance was estimated based on the average value [58]. For each search of hyperparameter combinations, a 10-fold validation result was used to evaluate the optimal value in the hyperparameter combination.

## 3. Discussion

### 3.1. Model Evaluation

The *R*^2^, RMSE, and MAE were selected as performance indicators to evaluate the accuracy of the ML model [59]. Equation (12) shows the calculation methods for obtaining relevant evaluation indicators, and the calculation results are shown in Table 2 and Table 3.

(12) $$R^{2}=1-\frac{\sum_{i=1}^{n}{\left({\hat{y}}_{i}-y_{i}\right)}^{2}}{\sum_{i=1}^{n}{\left({\bar{y}}_{i}-y_{i}\right)}^{2}}\;RMSE=\sqrt{\frac{\sum_{i=1}^{n}{\left({\hat{y}}_{i}-y_{i}\right)}^{2}}{n}}\;MAE=\frac{1}{n}\sum_{i=1}^{n}\left|{\hat{y}}_{i}-y_{i}\right|$$

### 3.2. Prediction Comparison of Models

**Figure 5:**
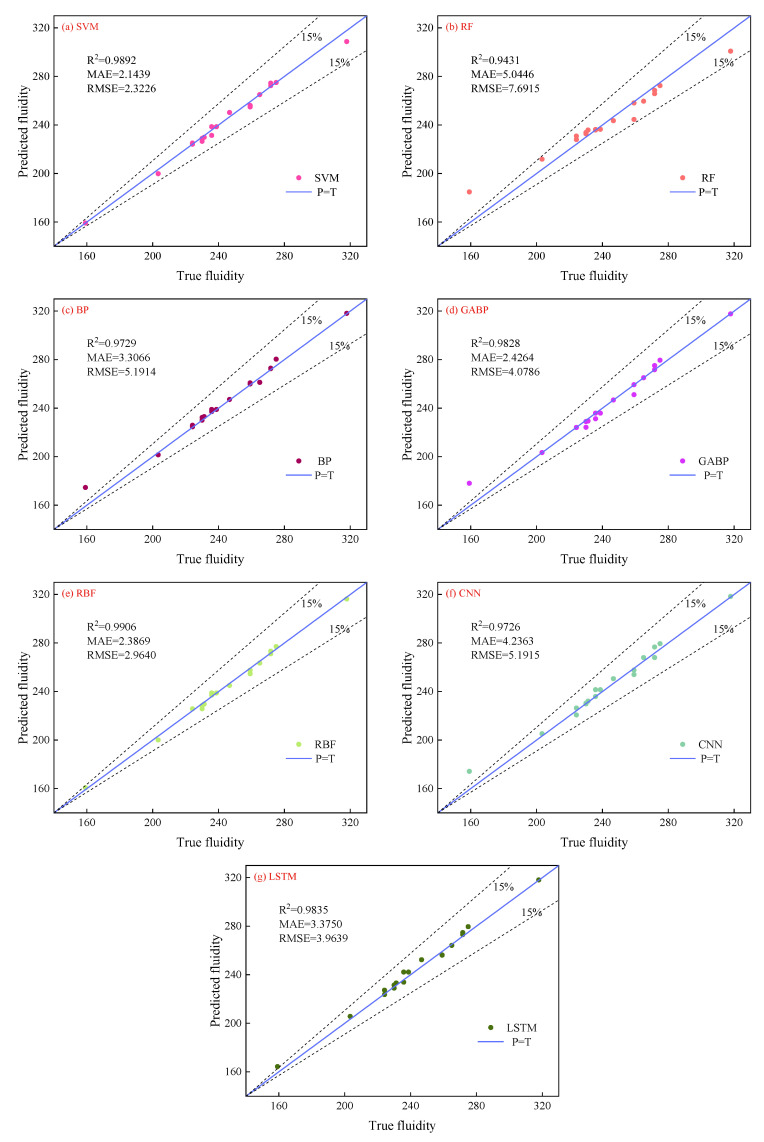
The deviation between the predicted value and the real value of different model fluidity.

**Figure 6:**
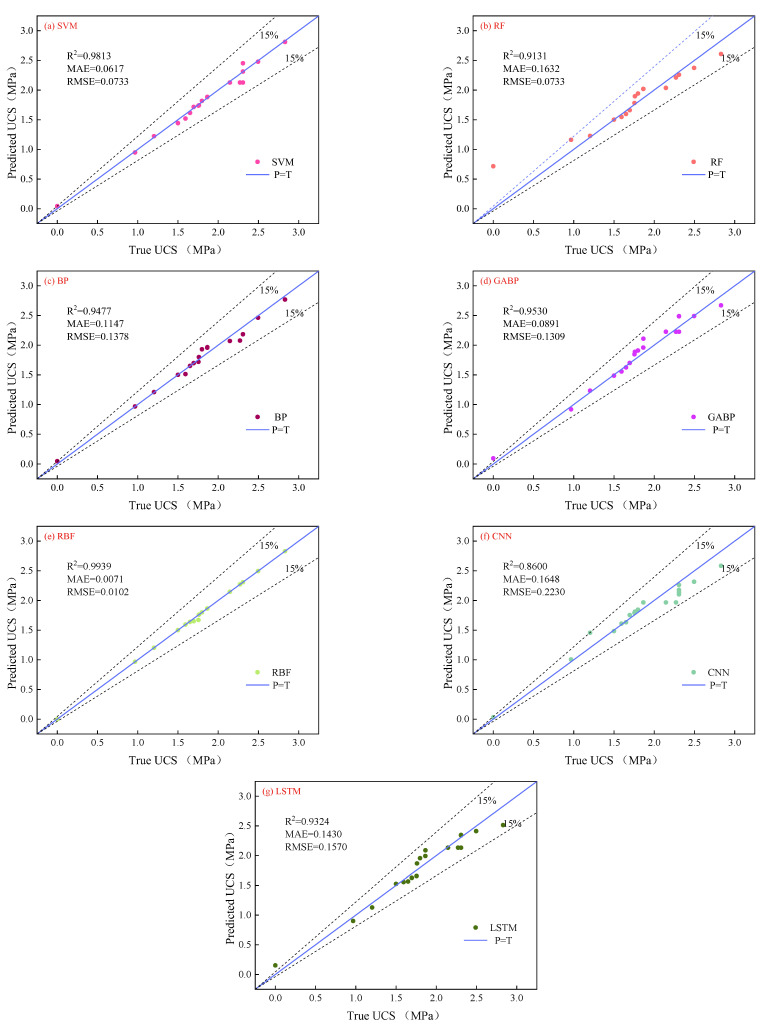
The deviation between the predicted value and the real value of different model UCS.

Figure 5 and Figure 6 show the comparison of values predicted by seven ML models against true fluidity and UCS.

Regarding fluidity, the SVM, GABP, RBF, and LSTM methods perform well in established ML model algorithms, with an *R*^2^ of 0.99 between predicted and true values. The *R*^2^ values of the other models also exceeded 0.90, with most predicted values within 15% of the perfectly fitted curve’s adaptation region. This finding indicates the feasibility of predicting the fluidity of CPB using ML model algorithms. Additionally, the RMSE and MAE of the SVM reach the minimum values of 2.32 and 2.14, respectively. The RBF model also performed relatively well, with RMSE and MAE values of 2.96 and 2.39, respectively. However, since RBF has the highest *R*^2^ and smaller differences between the training and testing sets compared to SVM, it can be considered to have the best predictive performance for fluidity. When the fluidity is 159.17 mm, the predicted values of the RF, BP, GABP, and CNN models significantly differ from the actual values, indicating that the accuracy of these models in predicting the minimum data values needs improvement.

Regarding compressive strength, the RBF method shows optimal performance among the seven ML model algorithms, with an *R*^2^ of 0.99. The prediction accuracy of the SVM is second only to the RBF method, with an *R*^2^ close to 0.99, while the *R*^2^ values of the RF, BP, GABP, and LSTM models range between 0.90 and 0.95. Currently, CNN performs poorly with an *R*^2^ of 0.86. When the intensity is 0, the predicted values of the RF and LSTM models are greater than the true values, indicating poor prediction of minimum values. During the training process, the RMSE and MAE obtained by the RBF model were both 0.00, and the *R*^2^ was 1. Additionally, the small difference between the training and testing sets verifies that the RBF method achieved the optimal prediction of UCS.

In summary, the RBF model has demonstrated superiority in predicting both fluidity and intensity, primarily due to its use of the Gaussian function as a kernel function. This function enables a non-linear mapping between the network’s input and output while maintaining a linear relationship with the adjustment parameters. Consequently, this method can directly solve the network’s weights using linear equations, enhancing learning speed and avoiding local optima [60,61].

**Figure 7:**
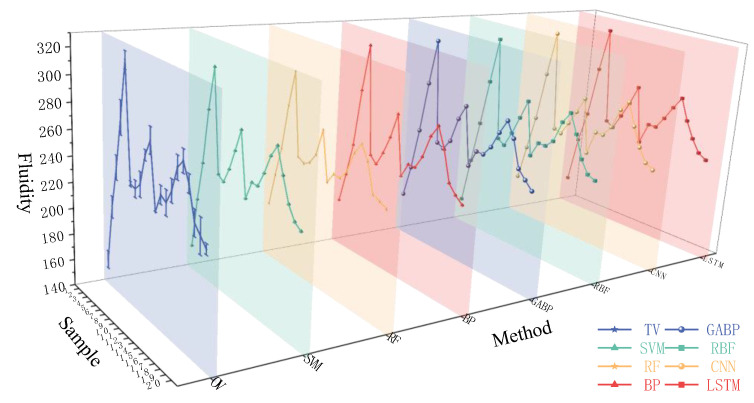
True value and predicted value of fluidity.

**Figure 8:**
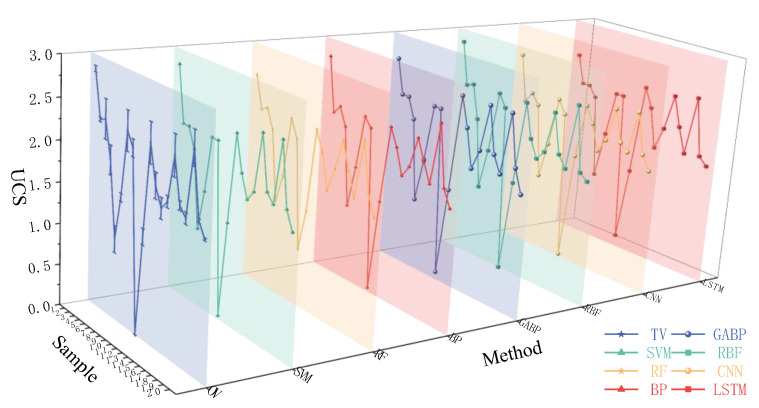
True value and predicted value of UCS.

Figure 7 and Figure 8 illustrate the differences between the calculated values of the seven ML models and the true values (TV). The graphs show that the models predict the median part of the data well but diverge significantly when predicting maximum/minimum values, which is influenced by the models themselves and their parameters. Table 4 lists ML models for predicting concrete performance, with *R*^2^ values ranging from 0.96 to 0.98. The *R*^2^ values of the ML models used in this study mostly range between 0.90 and 0.99, with the RBF model achieving the highest *R*^2^ of 0.99.

## 4. Dynamic Prediction on UCS

In practical engineering, the date when the filling material reaches the specified strength (2 MPa) is critical. Therefore, establishing a strong dynamic growth model using ML is crucial.

### 4.1. Strength Growth Prediction: Combination and Feedback

Generally, selecting a suitable mathematical parser for ML models is a key step in predicting the strength growth of filling materials. Assuming there is a vector, that is:

(13) $$\vec{\alpha }=(\alpha_{1},\alpha_{2},\alpha_{3})\;\alpha_{i}=\frac{t_{i}}{t_{4}}$$

where $t_{i}.i=1,2,3$ is the 3-day, 7-day, and 14-day strengths; $t_{4}$ is the 28-day strength.

**Figure 9:**
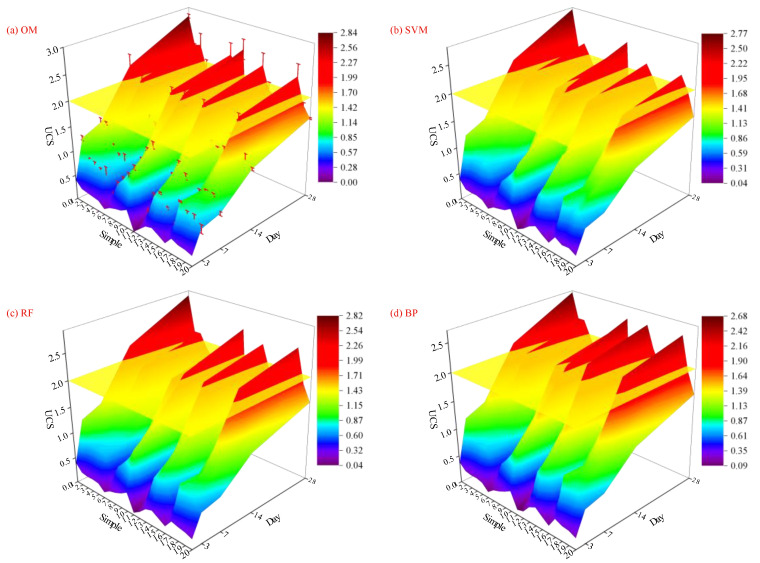

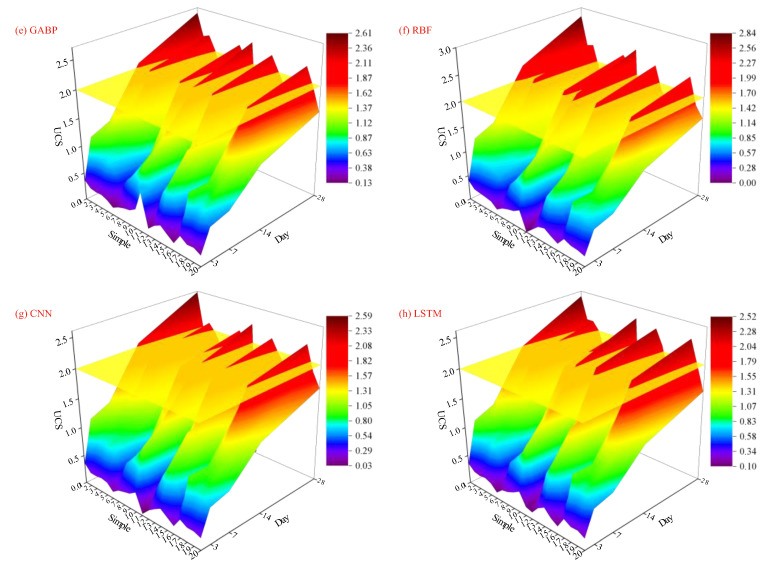
Curing ages to strength threshold predicted by different models.

Equation (13) was utilized to map the predicted 28-day strength of the backfilling material to 3-day, 7-day, and 14-day intervals and plot the growth curve of backfilling material strength with curing age. Due to the small horizontal time variation of the sample and the large number of time nodes, no fitting was performed on the time scale, and a linear model was adopted. A linear interpolation method was utilized to fit the spatial surface regions between samples, and the growth surfaces of strength predicted by each model are shown in Figure 9. The yellow plane in the figure represents a UCS of 2 MPa. Table 5 shows the curing age corresponding to the sample reaching 2 MPa (rounded up).

It was found from the comparison that BP and SVM models both show a lag in predicting the growth in strength of filling materials that could reach 2 MPa within 28 days, with the BP model exhibiting a more obvious lag. In the original strength growth model (OM), the filling materials M3 and M9 can reach 2 MPa on the 14th day and 22nd day. However, BP reaches 2 MPa on the 17th day and 27th day, and the lag becomes more pronounced as the strength of the filling material decreases. The results indicate that BP’s predicted strength of the filling material is smaller than the original value. The error is relatively large at this time, but the filling material is in a safer state. GABP, RF, and LSTM also have lagged in predicting the strength of filling materials that could reach 2 MPa, while they show overestimated predictions in the strength (an advancing trend in curing age) of filling materials that do not reach but are close to 2 MPa. The 28-day strength of M14 in OM is 1.87 MPa, but GABP, RF, and LSTM show that M14 reaches 2 MPa on the 26th day, 28th day, and 26th day, respectively. The results indicate that the strengths of filling materials predicted by the three models are higher than the original values, resulting in larger errors and a situation dangerous to filling materials. CNN exhibits a more obvious lag: M2 and M9 in OM reach 2 MPa on the 22nd day, but at this time, the 28-day strength predicted by CNN is only 1.97 MPa, which also indicates that the accuracy of the CNN model is the lowest. RBF shows an advantage, with the predicted values remaining consistent with the original values of the samples, and the fitting degree of the intensity growth surface reaches its optimal level.

### 4.2. RBF-Based Coupling Process of Mix Ratio and Curing Age

**Figure 10:**
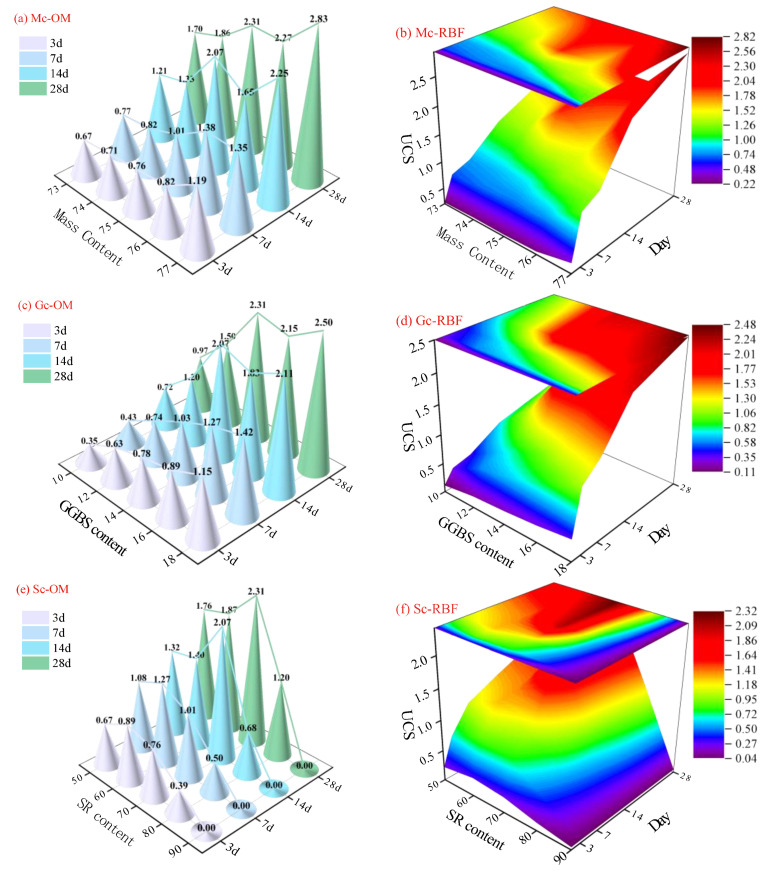

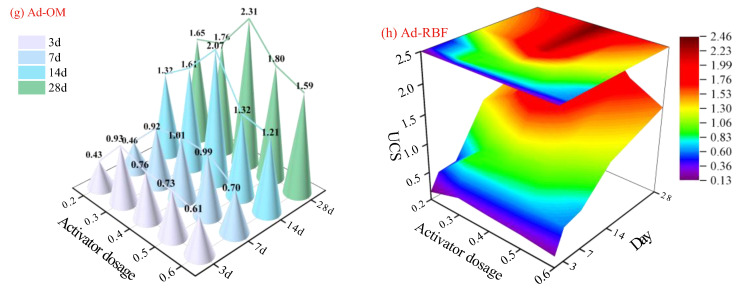
True UCS and RBF growth prediction models at different mix ratios.

Figure 10 shows the actual strength and RBF-predicted strength of the filling material under the coupling effects of different formulations (mass concentration, slag content, SR content, and activator content) and curing age. It is found that the value predicted by the RBF-coupled model shows a strong correlation with the actual values, meaning the predicted value increases synchronously with the rise of mass concentration and slag content. The strength of the filling body reaches its maximum when the content of SR is 70%, and the content of the activator is 0.4. In addition, the surface of the RBF-coupled model has the smallest angle between the tangent and the XY plane at this point, which also indicates that the strength of the filling material reaches its maximum at that point. In summary, the establishment of the RBF-coupled model helps to understand the growth pattern of the strength of filling materials under different mix ratios. The model’s prediction has the greatest fit with the true strength of the filling material, significantly reducing the number of experiments. The establishment of the RBF-coupled model helps to predict the mix ratio and curing age of the filling material; in other words, when the mix ratio is determined, the curing age at which the filling material reaches the specified strength can be predicted, providing a reliable theoretical basis for the safe operation of filling material. Conversely, when the interval of curing age is determined, multiple mix ratios can also be found to prepare the filling material. The optimal mix ratio can be determined based on the ratio between existing solid wastes, thereby making solid waste management more rational. Thus, it can be seen the establishment of the RBF model enables filling materials to be more reliably and efficiently applied in engineering practice, providing new ideas for the application of filling materials in engineering practice.

## 5. Conclusions

(1) Since the database established by the research institute contains a significant amount of data with a broad range, the ML model can learn more complex relationships and patterns within the data. This capability enhances its predictive performance and generalization ability.
(2) By comparing the predictive performance of seven ML models, it was found that the RBF model achieved the best prediction for the strength of the filling material, with all *R*^2^ values being 0.99. This was followed by SVM, with *R*^2^ values close to 0.99. The *R*^2^ values for RF, BP, GABP, CNN, and LSTM, except for the CNN prediction on UCS (*R*^2^ is 0.86), were all greater than 0.90. The performance of RF in predicting the maximum mechanical properties was poor.
(3) The RBF model can accurately predict the curing age at which the filling material reaches the specified strength. The BP, SVM, and CNN models exhibit delays in predicting the curing age, while the RF, GABP, and LSTM models show advances in predicting the curing age of filling materials to be close to 2 MPa.
(4) The ML technique has taken a meaningful step towards tailings management for the preparation of cemented paste fillers. The effect of material proportioning was mainly considered in this study using the ML technique, and therefore, further validation in other areas is warranted in the future. For example, further studies under different activators and multi-field coupling environments could be considered.

## Figures and Tables

**Table 1:** Summary of raw data.

Simple	Mass Content (%)	GGBS Content (%)	Activator Dosage	SR Content (%)	CS (%)	Fluidity	UCS
M1	77	14	0.4	70	30	159.17	2.83
M2	76	14	0.4	70	30	203.33	2.27
M3	75	14	0.4	70	30	235.83	2.31
M4	74	14	0.4	70	30	275.00	1.86
M5	73	14	0.4	70	30	317.83	1.70
M6	75	10	0.4	70	30	231.25	0.97
M7	75	12	0.4	70	30	230.00	1.50
M8	75	14	0.4	70	30	235.83	2.31
M9	75	16	0.4	70	30	259.17	2.15
M10	75	18	0.4	70	30	271.67	2.50
M11	75	14	0.4	90	10	224.16	0.00
M12	75	14	0.4	80	20	238.75	1.20
M13	75	14	0.4	70	30	235.83	2.31
M14	75	14	0.4	60	40	246.67	1.87
M15	75	14	0.4	50	50	265.00	1.76
M16	75	14	0.2	70	30	271.67	1.65
M17	75	14	0.3	70	30	259.17	1.76
M18	75	14	0.4	70	30	235.83	2.31
M19	75	14	0.5	70	30	230.00	1.80
M20	75	14	0.6	70	30	224.17	1.59

**Table 2:** Fluidity model and predictive evaluation value.

Technique	Train			Test		
	RMSE	*R* ^2^	MAE	RMSE	*R* ^2^	MAE
SVM	2.1513	0.9964	1.9888	3.0079	0.9603	2.7643
RF	8.6368	0.9419	5.5869	3.9101	0.9478	2.8753
BP	4.3567	0.9756	2.5110	8.5301	0.9620	6.4892
GABP	2.5228	0.9908	0.9365	10.3016	0.9508	8.3858
RBF	2.1437	0.9951	1.5911	6.2450	0.9726	5.5699
CNN	5.2180	0.9759	4.0448	5.0857	0.9594	5.0024
LSTM	1.5624	0.9977	1.2296	8.2988	0.9286	7.7467

**Table 3:** UCS model and predictive evaluation value.

Technique	Train			Test		
	RMSE	*R* ^2^	MAE	RMSE	*R* ^2^	MAE
SVM	0.0313	0.9957	0.0242	0.2413	0.9236	0.2115
RF	0.1852	0.9169	0.1365	0.0750	0.8977	0.0652
BP	0.1472	0.9467	0.1212	0.1000	0.9518	0.0886
GABP	0.1397	0.9485	0.0911	0.0957	0.9710	0.0811
RBF	0.0205	0.9973	0.0168	0.0509	0.9696	0.0353
CNN	0.2428	0.8566	0.1822	0.1438	0.8735	0.0954
LSTM	0.1707	0.9288	0.1582	0.1024	0.9470	0.0822

**Table 4:** Advantages of this study compared with other similar studies.

Study	Input Date	Output Date	Method	Achievements
[62]	Compressive strength of foamed concrete	Compressive strength	GBT	0.977 (*R*)
[63]	RHA concrete	Compressive strength	ANN-LM	0.9797 (*R*^2^)
[64]	Cement paste	Creep modulus	DSNN	Higher than 0.96 (*R*^2^)
This study	cemented tailings backfill	Fluidity and Compressive strength	SVM, RF, BP, GABP, RBF, CNN, LSTM	0.99 (*R*^2^)

**Table 5:** Curing ages to strength threshold predicted by different models.

Simple	OM	SVM	RF	BP	GABP	RBF	CNN	LSTM
M1	13	13	14	13	14	13	14	15
M2	22	25	24	27	23	22	--	25
M3	14	20	14	17	15	14	22	20
M4	--	--	28	--	--	--	--	--
M8	14	20	14	17	15	14	20	20
M9	22	23	27	25	19	22	--	22
M10	13	14	14	14	14	13	16	14
M13	14	14	14	17	13	14	17	14
M14	--	--	28	--	26	--	--	26
M18	14	14	14	17	14	14	14	14

## Data Availability

The original contributions presented in the study are included in the article, further inquiries can be directed to the corresponding author.
